# Larvicidal and Oviposition Activity of Commercial Essential Oils of *Abies sibirica* Ledeb., *Pogostemon cablin* (Blanco) Benth., *Juniperus communis* L. and Their Combinations Against *Aedes aegypti*

**DOI:** 10.3390/molecules29245921

**Published:** 2024-12-15

**Authors:** Júlio César Ribeiro de Oliveira Farias de Aguiar, Ana Carla da Silva, Eduarda Florêncio Santos, Gilson José da Silva Gomes Vieira, Liderlanio de Almeida Araújo, José Jorge Almeida de Andrade, Wevertton Marllon Anselmo, Suyana Karolyne Lino da Rocha, Fábio Henrique Galdino dos Santos, Camila Caroline Lopes Arruda, Caroline Francisca de Oliveira Albuquerque, Libna Larissa Monteiro Claudino, Priscila Soares da Silva, Danilo Gustavo Rodrigues Silva, João Vitor Castro Aguiar, Bruno Oliveira de Veras, Daniela Maria do Amaral Ferraz Navarro

**Affiliations:** 1 Department of Chemistry, Center for Exact and Natural Sciences, Federal University of Pernambuco, Recife 50670-901, Brazileduarda.florencio@ufpe.br (E.F.S.); gilson.vieira@ufpe.br (G.J.d.S.G.V.); liderlanioalmeida@gmail.com (L.d.A.A.); jorge.almeida@ufpe.br (J.J.A.d.A.); wevertton.anselmo@afogados.ifpe.edu.br (W.M.A.); suyanarocha@hotmail.com (S.K.L.d.R.); fabio.henriquegaldino@ufpe.br (F.H.G.d.S.); camila.lopesarruda@ufpe.br (C.C.L.A.); oliveira.albuquerque@ufpe.br (C.F.d.O.A.); libna.monteiro@ufpe.br (L.L.M.C.); priscila.soaress@ufpe.br (P.S.d.S.); danilo.gustavo@ufpe.br (D.G.R.S.); castro.aguiar@ufpe.br (J.V.C.A.); 2 Doctorate in Tropical Medicine, Laboratory of Microbiology, Federal University of Pernambuco, Recife 50670-901, Brazil; bruno.overas@ufpe.br

**Keywords:** larvicide, oviposition, deterrent, dengue, arboviruses, terpenes, natural products, bioassays

## Abstract

*Aedes aegypti* is a vector responsible for the transmission of various arboviruses and is considered by the World Health Organization to be one of the main public health problems in the world. This study evaluated the larvicidal and oviposition activity of essential oils from *Abies sibirica*, *Pogostemon cablin* and *Juniperus communis* and their formulations. Chromatographic analysis by GCMS identified a total of 28, 52 and 18 compounds for the oils of the species *A. sibirica*, *J. communis* and *P. cablin*, respectively. The larvicidal bioassays showed an LC_50_ of 67.53 ppm, 92.45 ppm and 35.95 ppm, respectively, for *A. sibirica* (A), *J. communis* (J) and *P. cablin* (P) as well as their binary (J + P, 39.50 ppm; A + P, 51.64 ppm) and ternary (A + J + P, 66.99 ppm) formulations. These oils and formulations also showed deterrent activity at the larvicidal concentrations tested (*A. sibirica*: OAI: −0.41; *J. communis*: OAI: −0.31; *P. cablin*: OAI: −0.62; A + J + P: −0.30; A + P: −0.68; A + J: −0.29; and J + P: −0.30). The oils and their formulations are a potential larvicidal source for mitigating the proliferation of diseases by this vector.

## 1. Introduction

*Aedes aegypti* (Linnaeus, 1762) is a vector responsible for transmitting arboviruses such as chikungunya, dengue, Zica virus and yellow fever, which affect millions of people around the world [1,2]. These diseases cause billions of dollars in losses to local economies, as well as serious health consequences, such as permanent damage to the brain formation of newborn babies (microcephaly) [3]. This mosquito has adapted well to urban centers and countries with hot and humid climates and large populations, and is considered by the World Health Organization (WHO) to be one of the main public health problems in the world [4,5]. According to the WHO, it is estimated that between 100 and 400 million infections by these arboviruses occur every year and that one death occurs every 12 min, threatening 4.2 billion people in 128 countries, with dengue being endemic in more than 100 countries [6].

Despite huge investments in programs to combat the vector, epidemic outbreaks of arboviruses are recurrent, since the number of mosquitoes immune to conventional insecticides tends to increase due to insect resistance mechanisms [7,8,9]. Even with the development of new technologies and research into the manufacture of vaccines against these arboviruses, it will still take a long time for effective and safe vaccines to emerge that can be used on a global scale and distributed to the entire population [10]. Thus, there is an interest in using chemical compounds of natural origin that can be effective at any stage of the vector’s life cycle in order to reduce the growing number of infections [11,12,13].

In this respect, the use of essential oils extracted from plants has gained importance because they have various properties against insects. For example, they can attract them, drive them away, hinder their development and even kill them, with terpenes being the main chemical constituents responsible for their insecticidal action [14,15]. These oils have various applications, such as in the food, pharmaceutical and cosmetics industries. They are less toxic to humans and other non-target animals, are not bioaccumulative, are easy to acquire and handle and have a range of biological activities that are economically viable [16].

Formulations made up of numerous possible combinations of oils or their main compounds make it possible for multiple mechanisms of insecticidal action to act simultaneously and therefore offer less likelihood of cross-resistance developing in the control of vectors and pests, as well as being less toxic than the use of just one main compound in isolation [17,18,19].

Among the various families that have chemical compounds with insecticidal potential, the Pinaceae, Cupressaceae and Lamiaceae families and their respective representatives stand out, such as the species *Abies sibirica* Ledeb., *Juniperus communis* L. and *Pogostemon cablin* (Blanco) Benth., which despite having various applications such as antifungal [20], healing [21], antioxidant [22], antiviral [23] and antibacterial [24] activities, in addition to the preservation of foodstuffs [25], have been little explored in terms of their action against *A. aegypti*. Therefore, the aim of this study was to evaluate the larvicidal and oviposition potential of the essential oils of these plants, alone and in formulations with equal proportions of these oils.

## 2. Results

### 2.1. Characterization of the Essential Oils of A. sibirica, J. communis and P. cablin

The essential oils of *A. sibirica*, *J. communis* and *P. cablin* were described by gas chromatography using two types of detectors where mass spectrometry was used to identify the oil constituents while flame ionization detection was used to quantify them. The constituents identified can be seen in Table 1 and the chromatograms in the Supplementary Material, Figures S1–S3, respectively. Also available in the Supplementary Material are the raw data from the GCMS spectra of each essential oil.

The chromatographic analysis revealed a total of 28, 52 and 18 compounds identified for the species *A. sibirica*, *J. communis* and *P. cablin*, respectively. The major constituents for *A. sibirica* are the monoterpenes δ-3-carene (12.65%) and camphene (20.83%) and the bicyclic monoterpene bornyl acetate (25.89%); for *J. communis*, they are the monoterpenes myrcene (14.64%), sabinene (18.42%) and α-pinene (24.73%); and for *P. cablin*, the sesquiterpenes α-guaiene (17.22%), α-bulnesene (20.40%) and patchouli alcohol (27.62%). It can be seen that monoterpenes are the most abundant compounds for *A. sibirica* (96.21%) and *J. communis* (79.29%), while for *P. cablin* most of the compounds are sesquiterpenes (92.74%). It is also possible to observe that the monoterpenes α-pinene and β-pinene and the sesquiterpene *(E)*-caryophyllene are present in the chemical composition of all three oils, and these compounds show larvicidal and oviposition deterrent action in the literature for larvicidal and oviposition activities against *Aedes aegypti* larvae and mosquitoes [15,26,27].

### 2.2. Larvicidal Activity

The results obtained can be seen in Table 2 and in the Supplementary Material Tables S1–S7. The tables in the Supplementary Material contain the concentration ranges tested, the total number of larvae used in the bioassays, and the total number killed at each concentration, as well as the percentage of mortality. Table 2 shows that the essential oils of *A. sibirica* (CL_50_ = 67.53 ppm), *J. communis* (CL_50_ = 92.45 ppm) and *P. cablin* (CL_50_ = 35.95 ppm) and their binary or tertiary formulations have a CL_50_ value below 100 ppm, except for the binary formulation formed by the oils of *A. sibirica* + *J. communis* (CL_50_ = 363.89 ppm). However, of these oils and formulations, those containing the *P. cablin* EO (*P. cablin* > *A. sibirica* = *J. communis*) stand out, as well as their binary formulations (J + P, 39.50 ppm; A + P, 51.64 ppm; A + J, 363.89 ppm) and ternary formulations (A + J + P, 66.99 ppm). Thus, among the essential oils tested separately, *P. cablin* oil was the most bioactive in causing the death of the insect’s larval stage. Among the formulations, those with the presence of *J. communis* and *P. cablin* (J + P) stood out, the latter being the most bioactive in promoting the mortality of *A. aegypti* larvae (J + P > A + P = J + P + A > J + A). Thus, *P. cablin* oil and its J + P formulation were the most bioactive products for larvicidal activity in this study.

The addition of *P. cablin* oil to *A. sibirica* oil, for example, favored the larvicidal action of this oil isolated from *A. sibirica*, since the A + P formulation decreased by 15.89 ppm (23.53%) to obtain the same effect of 50% mortality of the *Ae. aegypti* larval population. It is also believed that the greatest effect on mortality of the A + J formulation is due to a general increase in the sesquiterpene content, since the main constituents of *A. sibirica* oil are the monoterpenes δ-3-carene (12.65%) and camphene (20.83%) and the bicyclic monoterpene bornyl acetate (25.89%), while *P. cablin* oil is composed of the sesquiterpenes α-guaiene (17.22%), α-bulnesene (20.40%) and patchouli alcohol (27.62%) as its main constituents. Regarding the J + A formulation, it is believed that its larvicidal action was impaired due to the lower percentages of sesquiterpenes in the *A. sibirica* and *J. communis* oils.

### 2.3. Oviposition Activity

**Figure 1:**
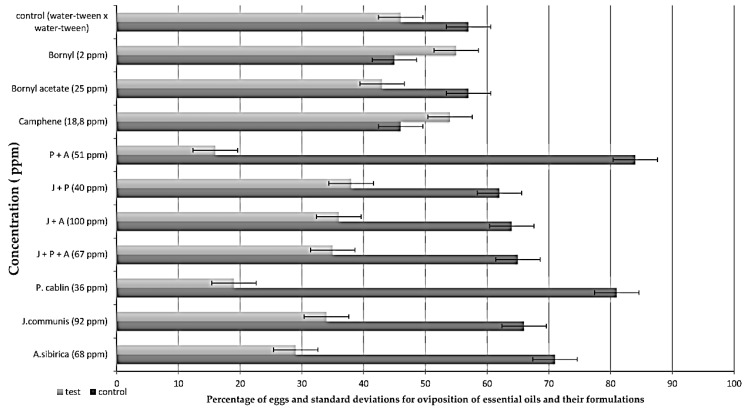
Histogram with the average percentage of eggs deposited in the oviposition bioassay and the respective deviation for control solutions, essential oils and their formulations.

Oviposition bioassays were also carried out at LC_50_ concentrations for the essential oils and some isolated compounds and formulations on female *A. aegypti* mosquitoes. The results obtained can be seen in Table 3 and Figure 1 as a function of the percentage number of eggs. Further results can be seen in the Supplementary Material Tables S8–S18.

As shown in Table 3 and Figure 1, there was a significant preference for the controls over the tests for all the oviposition bioassays carried out. It can also be seen that the oviposition bioassay for the control alone showed no significant difference (*p*-value: 0.213, N: 4435) in the choice of female, thus excluding possible interference from the work environment. The tests carried out with camphene, bornyl acetate and bornyl showed that the females had no preference for these compounds when compared to the control. Another parameter used to evaluate the results obtained is the oviposition activity index (OAI). This index indicates that the compound is considered attractive when it is positive and greater than +0.30, while a compound with a negative index of less than −0.30 is considered repellent. And between this range of +0.30 and −0.30 it has no oviposition activity [28,29]. Most of the essential oils and formulations tested have oviposition-deterrent activity with OAIs close to and greater than −0.30, confirming the females’ preference for oviposition in the controls.

## 3. Discussion

### 3.1. Characterization of the Essential Oils of A. sibirica, J. communis and P. cablin

In the literature, differences in the composition or chemical ratio of the oils are related to the seasonal abiotic and biotic factors to which the plants are subjected. Some of these factors are the vegetative cycles of the plants, the process of obtaining the essential oil, the environment in which a species grows, the type of cultivation, the temperature, relative humidity, the amount of water and nutrients in the soil, and the storage and conservation of the oil, among other factors [30,31]. However, the major compounds found in *A. sibirica* are in line with those reported in the literature, where the predominant compounds are monoterpenes and sesquiterpenes such as α-pinene, bornyl acetate, camphene and carene. These compounds and their derivatives are responsible for various biological activities [32]. The hydrodistillation yield for this species can vary between 1.42 and 2.97% [33]. The chemical profile of *J. communis* was similar to that reported in the literature for the major compounds α-pinene, sabinene, myrcene and limonene [34], with yields varying between 0.24 and 1.64% [35,36]. The essential oil of *P. cablin* is rich in sesquiterpenes, including β-elemene, α-patchoulene, α-guaiene, β-caryophyllene, β-patchoulene, α-bulnesene and patchouli alcohol [37], with yields ranging from 1.40 to 2.80% [38,39].

The chemical identification of *A. sibirica* oil obtained experimentally corroborates that found in the literature. For example, in the study by Polyakov and collaborators (2014) [40] the same major constituents were found: bornyl acetate (34.2%) and camphene (17.5%), in relatively similar concentrations. Similarly, the studies by Sundaresan and collaborators (2009) [41] on the essential oil of *P. cablin* obtained by hydrodistillation with a yield of 0.3% found the same major constituents in slightly higher percentages: patchouli alcohol (23.2%) and α-guaiene (14.6%). The composition reported for the studies by Höferl and collaborators (2014) [42] on the essential oil of *J. communis* also shows the same three major components, α-pinene (51.4%), myrcene (8.3%) and sabinene (5.8%), but with different quantities, where the largest component accounts for more than half of the essential oil’s chemical composition. Thus, even at different percentages, these oils maintain the same major constituents, which are found to be characteristic of the chemical profile of each species [43,44].

### 3.2. Larvicidal Activity

As reported in the literature, essential oils that show larvicidal activity at a lethal concentration of up to 50 ppm are considered very bioactive, while those that show activity in the LC_50_ range between 50 and 100 ppm can be considered active and those with a LC_50_ greater than 100 ppm are considered inactive [15,45]. Thus, the essential oil of *P. cablin* (35.95 ppm) is classified as very bioactive, followed by the oils of *A. sibirica* (67.53 ppm) and *J. communis* (92.45 ppm), classified as active. Among the binary oil formulations, the combination of *J. communis* and *P. cablin* essential oils (39.50 ppm) can be classified as very bioactive, followed by the combination of *A. sibirica* and *P. cablin* (51.64 ppm) and the three combined oils *A. sibirica*, *J. communis* and *P. cablin* (66.99 ppm), whose results show these combinations as active, while the combination of *A. sibirica* and *J. communis* essential oils (363.89 ppm) was inactive.

As for the binary and tertiary formulations of the oils, it can be seen that the *P. cablin* oil seems to increase the results of the larvicidal activities obtained for the formulations containing this oil, since the only formulation in which this oil is not present (*A. sibirica* and *J. communis*) showed an LC_50_ classified as inactive, with a result exceeding 100 ppm, suggesting an antagonistic action for the combination of these essential oils. The results obtained for the essential oil of *P. cablin*, alone or in combination, can be explained by the fact that its major compounds belong to the sesquiterpene class.

Studies in the literature have reported greater larvicidal activity of sesquiterpenes compared to monoterpenes for *A. aegypti* mosquito larvae, due to the lipophilic property of terpenes (increased transmembrane absorption by the body, which can cause toxic effects), with sesquiterpenes being the most active for this property [46]. Among the terpenes described in the literature and present in the chemical constitution of the oils studied with larvicidal activity are the monoterpenes α-pinene (LC_50_ from 15.4 to 79.1 ppm), β-pinene (LC_50_ from 12.1 to 27.69 ppm) and limonene (LC_50_ from 12.01 to 19.4 ppm), oxygenated monoterpenes such as bornyl (LC_50_ from 43.5 to 94.9 ppm), as well as the sesquiterpenes β-caryophyllene (LC_50_ = 88.3 ppm), δ-cadinene (LC_50_ = 9.03 ppm) and α-humulene (LC_50_ = 30.89 ppm) [18,47].

Drawing a parallel between the lethal concentrations of the essential oils of *A. sibirica* and *J. communis* in relation to other essential oils from Brazilian plants, we can consider close values for the larvicidal activity against *A. aegypti* for the plants *Ocimum gratissimum*, *Lippia sidoides*, *Cymbopogon citratus* and *Ocimum americanum*. While compared to the essential oils of *Eugenia candolleana*, *Eugenia piauhiensis*, *Cordia curassavica*, *Myrcia erythroxylon*, *Alpinia zerumbet*, *Psidium myrsinites*, *Citrus limonia*, *Siparuna camporum*, *Citrus sinensis*, *Lippia gracilis*, *Syzygium jambolanum*, *Cymbopogon winterianus*, *Baccharis reticularia*, *Croton tetradenius*, *Schinus terebinthifolia*, *Carapa guianensis*, *Commiphora leptophloeos* and *Hyptis suaveolens*, the LC_50_ values found for *A. sibirica* and *J. communis* in this study were better than many of the literature results presented (Table 4). On the other hand, the essential oil of *P. cablin* showed excellent larvicidal activity (Table 4), close to the larvicidal activities of plants of the genus *Piper* such as *P. gaudichaudianum* and *P. marginatum* and better than the species *P. arboreum* and *P. crassinervium* for the Rockefeller strains of *A. aegypti* tested [42]. The genus *Piper* has monoterpenes and sesquiterpenes in its composition, with larvicidal action already reported in the literature for the larvae of *A. aegypti* mosquitoes [48,49].

When comparing the larvicidal results of this study with data available in the literature, no studies were found on the insecticidal activity of *A. sibirica* oil on *A. aegypti*. The few studies found of *A. sibirica* oil focused on the antibacterial activity against strains of *Escherichia coli*, *Staphylococcus aureus* and *Pseudomonas aeruginosa* [55] and insecticidal activity against other insects such as peach aphids (*Myzus persicae* Sulz) [20,56].

However, there are studies in the literature on the mosquito larvicidal activities of essential oils from the Pinaceae family, conifers, such as *Pinus longifolia* oil, whose main constituents were α-terpineol (12.89%), eugenyl acetate (1.76%), eugenol (3.14%), isoeugenol (4.93%) and camphor (1.26%), and larvicidal activity against the larvae of *A. aegypti* (LC_50_: 82.1 ppm), *Culex quinquefasciatus* (LC_50_: 85.7 ppm) and *Anopheles culicifacies* (LC_50_: 112.6 ppm) [57]. Pavela and collaborators (2021) [58] also carried out studies to evaluate the insecticidal activity of the essential oils of conifers, including the Pinaceae family (*Picea abies*, *Pinus halepensis* and *Pinus sylvestris*) and the Cupressaceae family (*Juniperus chinensis* cv. ‘Stricta’, *J*. *communis* and *J*. *pfitzeriana*) against three important insect species: *Spodoptera littoralis*, *Culex quinquefasciatus* and *Musca domestica*. The main constituents of these species were monoterpenes and sesquiterpenes such as α-pinene (26.6 to 32.8%), sabinene (17.0 to 22.1), β-phellandrene (5.0 to 7.6%) and terpinen4-ol (5.8 to 7.4%) for the genus *Juniperus* and α-pinene (9.5 to 25.7%) and limonene (13.4 to 15.6%) for the species *Picea abies* and *Pinus sylvestris* (Pinaceae).

As for the oil of some of the species in the family Cupressaceae, such as *J. macropoda* Bois (LC_95_ = 171.3 ppm), *J. virginiana* L. (50 ppm) and *J. communis* L. (50 ppm), it is possible to find in the literature studies with significant mortality for the larval stage of *A. aegypti* [47]. However, many of these studies do not state an LC_50_ value, the larval stage or even the use of positive or negative controls to determine these values. There are also studies of the control of some vectors, such as *Aedes albopictus*, which reveal an LC_50_ of 55.5 ppm for the oil of *J. phoenicea*, whose main constituents are α-pinene (31.3%), δ-3-carene (12.5%), β-phellandrene (13.0%) and α-terpinyl acetate (12.5%) [59]. Studies of the control of *Anopheles arabiensis* indicate an LC_50_ of 10.82 ppm and a composition of sabinene (25.46%), DL-limonene (16.36%), β-myrcene (6.0%), bornyl acetate (5.18) and terpinen-4-ol (4.90%) for *J. virginiana* oil [60]. There are also larvicidal studies in the literature for *J. communis* oil with an LC_50_ of 163.6 ppm for *A. aegypti* where the main constituents were α-pinene (27.0%), α-terpinene (14.0%) and linalool (10.9%) [61]. It should be noted that for these studies mentioned above, the monoterpenes α-pinene and sabinene are the major compounds in each case, and these are also the two major compounds in the oil in this study (24.73 ± 2.29% α-pinene and 18.42 ± 1.58% sabinene). These levels are close to those found in the studies mentioned above, especially when it comes to the *A. aegypti* vector, but those studies recorded higher LC_50_ values than in this study.

For the oil of the species *P. cablin*, it is possible to find larvicidal values in the literature for *A. aegypti*, with the LC_50_ ranging from 25.14 to 46.40 ppm [39]. Santos and collaborators (2019) [62] also found a composition mainly of sesquiterpenes, such as seychellene (6.12%), α-bulnesene (4.11%), norpatchoulenol (5.72%), pogostol (6.33%) and patchouli alcohol (33.25%), and an LC_50_ of 28.43 ppm for *P. cablin* essential oil using *A. aegypti* larvae at the L3 stage. The present study carried out a larvicide test with L4 larvae, where the oil’s main substances were found to be α-bulnesene (20.40%) and patchouli alcohol (27.62%), and the LC_50_ was 35.95 ppm. The lower lethal concentration values found in these studies available in the literature are linked to the higher levels of sesquiterpenes, such as in patchouli alcohol. However, many of these studies used early larval stages, L2 and L3, while this study used the final stage, L4, the last stage before pupation, where ingestion of these larvicidal compounds no longer occurs [63].

Various studies have been carried out on the larvicidal activity of the essential oils of different plant species, where those with higher content of oxygenated sesquiterpenes, monoterpene hydrocarbons and phenylpropanoids have an LC_50_ < 100 ppm and are considered the most active. Among some of these outstanding specimens are those belonging to the families Myrtaceae, Apiaceae, Piperaceae, Lamiaceae, Rutaceae, Asteraceae, Meliaceae and Zingiberaceae [64]. Thus, the binary and ternary formulations based on the essential oils of *A. sibirica*, *J. communis* and *P. cablin* have their larvicidal action against *Aedes aegypti* larvae justified by the synergism of the presence of mono- and sesquiterpenes in the oils and their formulations.

### 3.3. Oviposition Activity

The choice of the area where the females will lay their eggs is made not only due to the availability of favorable environmental factors, but also due to the absence of threats to their development during the aquatic phase. To this end, females use strategies to assess the safety of egg laying. The chemoreceptor system is a resource used to recognize volatile chemical compounds which can attract or repel females and interfere with the mosquitoes’ oviposition behavior [63,65]. When comparing the results with the literature, it can be seen that the essential oil of the species *P. cablin* had a 62% greater effect. These results were better than those recorded for the species *Piper marginatum* and *Croton rhamnifolioides*, where this effect was only 20% and 40%, respectively [66,67]. The essential oils of *A. sibirica* and *J. communis*, on the other hand, had a 42% and 32% greater oviposition deterrent effect on oviposition activity, respectively, than the controls, similar to the activity of the red and pink variants of *Alpinia purpurata*, where a 30% inhibition of eggs was observed compared to the control solution [68].

In the literature, there are some studies indicating that the presence of some mono- and sesquiterpenes such as limonene, caryophyllene oxide, α-pinene, β-pinene, δ-elemene, germacrene D, β-caryophyllene, camphene and γ-terpinene are responsible for the deterrent action against species of *Aedes* [69,70]. There are also studies of the repellent [61] and oviposition [59,71] activities of the essential oil of *J. communis*. The essential oil of *P. cablin* has been studied for its repellent activity against this vector [72], but not for oviposition, while no studies were found for the oviposition or repellent activity of *A. sibirica* oil. The use of these essential oils is therefore capable of hindering and reducing the oviposition of female mosquitoes of *A. aegypti*, thus contributing as a potential natural alternative to be used in the control of this vector.

One of the main advantages of using essential oils as natural insecticides is that they are eco-friendly [73] and have a low negative impact on non-target organisms. Moreover, they can be easily obtained and have a high yield. Essential oils are a sustainable source for extraction, especially in a context of growing insect resistance to synthetic pesticides. EO extraction raises the profile of the biodiversity of plants that are adapted to dry climates and are little studied, promotes the conservation of local flora and strengthens the economies of local communities, causing positive social and environmental impacts. On the other hand, its high volatility represents a challenge for the development of a commercial formulation. Thus, nanoencapsulation of this natural product could be an efficient way of promoting its greater durability in the environment over time.

## 4. Materials and Methods

### 4.1. Essential Oils and Formulations

The essential oils extracted by steam extraction from the leaves of *Abies sibirica*, the seed coats of *Juniperus communis* and the roots of *Pogostemon cablin* were purchased from the commercial supplier dōTERRA^®^ Cosméticos do Brasil Ltda (Barueri, São Paulo, Brazil). According to the supplier, these plants were grown in Siberia, Albania and Indonesia, and their yields are equal to 2.8%, 0.9% and 2.1%, respectively. The essential oils were formulated by adding equal quantities of oils in proportions of 1:1:1 or 1:1 (*v*/*v*) dissolved in distilled water with the aid of a surfactant, Tween 80^®^ (Sigma-Aldrich, St. Louis, MO, USA).

The commercial oil samples used were all pure. However, the bioassays were carried out with them diluted, where the dilution was made by weighing the mass needed to prepare the solution at the desired concentration (masses varying between 1 mg and 125 mg, see Supplementary Material, Tables S4–S19) and this mass was diluted in distilled water (varying between volumes of 50 and 250 mL) with the help of a co-solvent, Tween 80^©^, 3 drops maximum. The approximate mass of 3 drops of Tween 80^©^ (density of 1.06 g·mL^−1^) was 2.6 mg for a 50-mL volumetric flask, which corresponds to a concentration of 52 ppm or 0.24% (*v*/*v*), which is very low to cause mortality as observed experimentally in the negative control or even described in the literature by Kramer et al. (1983) [74] for the toxicity of Tween 80^©^ (CL50 = 8%, *v*/*v*). The formulations were prepared by adding equal parts of the pure essential oils to form the desired formulations in proportions of 1:1:1 or 1:1. For example, to form the ternary mixture of oils, 0.5 mL of each of these pure oils was taken, added to a vial and homogenized using a vortex at 600 rpm. After this, the biosensing was carried out by diluting the pure essential oils or formulating equal parts of the oils in distilled water and 3 drops of co-solvent (Tween 80^©^, surfactant).

### 4.2. Characterization of Essential Oils

The composition of each oil was analyzed in a gas chromatograph coupled (Agilent Technologies, Palo Alto, CA, USA) to a mass spectrometer (GCMS) equipped with an Agilent 5975C series GC/MSD quadrupole (Agilent Technologies, Palo Alto, CA, USA) and an Agilent J & W DB-5 unpolished fused silica capillary column (30 m × 0.25 mm with a film thickness of 0.25 μm) (Agilent Technologies, Palo Alto, CA, USA). A volume of 1 μL of hexane oil solution (100 ppm) was injected into an injector operated in split mode (50:1) at an injector temperature of 250 °C. The oven temperature was initially set at 40 °C for 2 min, followed by heating at 4 °C/min to 230 °C, where it remained for 5 min. The flow rate of the mobile phase, helium gas, was 1 mL·min^−1^ while maintaining a constant pressure of 7.0 psi. The MS source and quadrupole temperatures were set at 230 °C and 150 °C, respectively. Mass spectra were obtained at 70 eV (in EI mode) with a scan speed of 1.0 s for a range of *m/z* 35–350 [75,76].

The chemical constituents were identified by comparing the retention indices reported in the literature, obtained by co-injecting samples of the essential oil with a homologous series of hydrocarbon standards (C_9_–C_30_ alkane standard, Sigma Aldrich, Milwaukee, WI, USA) and calculated according to the equation of Van den Dool and Kratz (1963) [77]. Identification was also made by comparing the mass spectra with spectral data available in the literature [78,79] and those in the following library databases: MassFinder 4, Dr. Hochmuth Scientific Consulting, Hamburg, Germany; NIST08 Mass Spectral Library (ChemSW Inc., Fairfield, CA, USA); Wiley Registry ™ of Mass Spectral Relations 9th Edition (Wiley, Hoboken, NJ, USA).

The oils were quantified in a gas chromatograph with flame ionization detector (GC-FID) (Thermo Trace GC Ultra, Milan, Italy) at 250 °C and a VB-5 apolar column (Thermo trace GC ultra, 60 m × 0.25 mm internal diameter; film thickness 0.25 μm). A volume of 1.0 μL of hexane oil solution (100 ppm) was injected in triplicate in splitless mode and under the same conditions as described above for the GCMS [80].

### 4.3. Creation of A. aegypti Colony

All the bioassays were carried out with larvae and mosquitoes from the *A. aegypti* colony (Rockefeller strain) kept in the Bioassay Laboratory of the Department of Fundamental Chemistry at UFPE under ambient temperature conditions of 27 ± 1 °C and humidity of 75 ± 1%. Rearing began with the hatching of *A. aegypti* eggs in rectangular plastic trays. For this purpose, trays containing mosquito eggs were covered with a volume of distilled water to fully immerse them and small portions of crushed feed (Whiskas^®^) (Abreu e Lima, Pernambuco, Brazil) were added. As the larvae developed, the distilled water and food were renewed every two days. At the end of the larval stage and the beginning of the pupal stage, the remaining larvae and pupae were gathered in cages, placed in several 10 cm diameter glass beakers containing distilled water and feed to await the emergence of the winged stage of the mosquitoes. Once emerged, the mosquitoes were fed a 10% sucrose solution [81].

### 4.4. Larvicidal Bioassays

For each larvicide bioassay, 20 larvae at the L4 stage were added to 40 mL beakers containing 20 mL of solution at concentrations ranging from 20 to 100 ppm. A bioassay was also carried out with a negative control (distilled water and Tween 80^®^ only). A positive control was also carried out with a solution of temephos (LC_50_ = 3.30 ± 0.18 ppb). Larval mortality was counted after 24 and 48 h of exposure to the oil solutions and formulations. Larvae that did not respond to stimuli or presented morphological alterations, such as deformation, darkening and apparent motor difficulties, were considered dead [82]. Once the concentrations with a mortality rate of between 20 and 80% had been determined, further larvicide tests were repeated in triplicate to statistically determine the lethal concentration to kill 50% of the individuals. The LC_50_ was obtained using StatPlus (Probit) 6.2.5.0 statistical software at a 95% confidence level [81].

### 4.5. Oviposition Bioassays

The bioassays were carried out using a stock of 250 mL of control solution (just distilled water and Tween 80^®^) and the same volume of stock for the test solution at the LC_50_ concentrations. Ten pregnant females were distributed in eight clean cages measuring 33 × 21 × 30 cm. A volume of 25 mL of each stock solution was added to filter paper folded into a conical shape and placed in identified cups numbered 1 to 8. Each cage received a beaker with the test solution and a beaker with the control solution placed diagonally across the cages in opposite and alternating spaces [26].

This bioassay was carried out for 16 h without any light and under the same relative temperature and humidity conditions described for colony maintenance. After this scotophase, the number of eggs laid in the control and test cages was counted manually for each cage. The data obtained were statistically analyzed using MINITAB Release 14 and Student’s *t*-test, considering tests with a *p*-value < 0.05 to be significant. In addition, the oviposition activity index (OAI) was calculated (Equation (1)) [29]. The results can be seen in the Supplementary Material in Tables S9–S19.

(1) $$OAI=\frac{\left({{NT}_{test}-NC}_{control}\right)}{\left({NT}_{test}+{NC}_{control}\right)}$$

where NC is the number of eggs in the control, NT is the number of eggs in the test and OAI is the oviposition activity index. The OAI value indicates whether a compound is attractive, deterrent or has no activity. OAI values in the −1 to −0.3 range, NC > NT, indicate that the female has chosen the sites of the control solutions (blanks) rather than the tests. While values in the range +1 to +0.3, NT > NC, indicate that the female has preferentially chosen the sites of the test solutions over the controls (whites). Furthermore, OAIs in the range −0.3 to +0.3 indicate that there is no preferential choice for any of the test or control solutions [28].

## 5. Conclusions

The essential oils of *A. sibirica*, *J. communis* and *P. cablin* and their binary and ternary formulations showed larvicidal and oviposition activity against *Aedes aegypti* larvae and mosquitoes, with the LC_50_ equal to 67.53 ppm for the EO of *A. sibirica*, 92.45 ppm for the EO of *J. communis* and 35.95 ppm for the EO of *P. cablin*. The LC_50_ values for their binary and ternary formulations were as follows: J + P, 39.50 ppm; A + P, 51.64 ppm; A + J, 363.89 ppm; and A + J + P, 66.99 ppm. Therefore, the essential oils of *A. sibirica*, *J. communis*, and *P. cablin* show larvicidal activity against *A. aegypti*, the latter being the most bioactive. The formulation with *J. communis* and *P. cablin* (J + P) was the most bioactive against *A. aegypti* larvae (J + P > A + P = J + P + A > J + A). Thus, *P. cablin* oil and the J + P formulation were the most bioactive products for larvicidal activity in this study. Regarding oviposition activity, both the oils and their formulations were shown to deter mosquito oviposition at the LC_50_ of larvicidal activity. Therefore, both essential oils and formulations are a promising source for the development of larvicidal and deterrent materials to mitigate the proliferation of diseases by this vector. They are an excellent insecticide alternative because they are extracted from renewable sources, do not present high toxicity to non-target animals and are biodegradable. Furthermore, they reduce the number of vector insects in the environment, thus being an ally of epidemiological, entomological and environmental surveillance and a market niche for the insecticide and repellent industries.

## 6. Patents

This work has intellectual property rights and has a national patent application for invention, utility model, certificate of addition of invention and entry into the national phase of the PCT with the National Institute of Industrial Property (INPI) under process number BR 10 2023 0248780.

## Figures and Tables

**Table 1:** Identification of the chemical constituents of the essential oils of *A. sibirica, P. cablin* and *J. communis.*

Chemical Constituent ^a^	CAS ^b^	RI		*A. Sibirica*		*P. Cablin*		*J. Communis*	
		Literature ^c^	Calculated ^d^	% ^e^	SD ^f^	% ^e^	SD ^f^	% ^e^	SD ^f^
Santene	529-16-8	884	882	3.47	0.07				
NI			889	0.07	0.01				
tricyclene	508-32-7	921	917	2.67	0.07			0.01	0.00
α-thujene	2867-05-2	924	924	0.01	0.00			2.36	0.21
α-pinene	80-56-8	932	930	13.26	0.20	0.19	0.04	24.73	2.29
camphene	79-92-5	946	944	20.83	1.19			0.25	0.10
NI			949					0.02	0.01
NI			961					0.05	0.01
NI			965	0.06	0.01				
sabinene	3387-41-5	969	970					18.42	1.58
β-pinene	127-91-3	974	970	2.86	0.09	0.22	0.04	2.10	0.17
myrcene	123-35-3	988	988	0.79	0.03			14.64	0.53
α-phellandrene	99-83-2	1002	999	0.17	0.00			0.24	0.04
δ-3-carene	13466-78-9	1008	1005	12.65	0.65			0.17	0.07
α-terpinene	99-86-5	1014	1011	0.09	0.01			1.38	0.29
ρ-cymene	99-87-6	1020	1019	0.10	0.01			0.75	0.08
limonene	138-86-3	1024	1024	9.70	0.23			5.67	0.25
1,8-cineole	470-82-6	1026	1025					0.01	0.00
*(E)-*β-ocimene	3338-55-4	1044	1044					0.01	0.00
γ-terpinene	99-85-4	1054	1053	0.17	0.02			2.49	0.38
*cis*-sabinene hydrate	17699-16-0	1065	1061					0.09	0.01
terpinolene	586-62-9	1086	1082	1.37	0.06			1.98	0.20
*trans*-sabinene hydrate	15826-82-1	1098	1091					0.08	0.02
linalool	78-70-6	1095	1095					0.08	0.01
NI			1097	0.05	0.01				
*cis*-ρ-menth-2-en-1-ol	29803-82-5	1118	1114					0.01	0.00
α-campholenal	4501-58-0	1122	1120					0.01	0.00
trans-sabinol	471-16-9	1137	1131					0.01	0.00
*trans*-limonene oxide	4959-35-7	1137	1133					0.01	0.00
NI			1140					0.06	0.02
camphor	76-22-2	1141	1137	0.21	0.03				
NI			1140	0.24	0.03				
borneol	507-70-0	1165	1159	1.82	0.17			0.01	0.00
terpinen-4-ol	562-74-3	1174	1171	0.09	0.01			3.10	0.44
NI			1179	0.05	0.01				
NI			1179					0.09	0.02
NI			1184	0.14	0.02				
α-terpineol	98-55-5	1186	1184					0.30	0.08
NI			1189					0.06	0.02
*cis*-piperitol	34350-53-3	1195	1201					0.01	0.00
citronellol	106-22-9	1223	1223					0.01	0.00
NI			1229					0.06	0.01
methyl ether thymol	1076-56-8	1232	1230	0.04	0.01				
NI			1238					0.03	0.01
NI			1248					0.08	0.01
bornyl acetate	76-49-3	1284	1283	25.89	1.77				
2-undecanone	112-12-9	1293	1289	0.02	0.01			0.04	0.01
methyl citronellate	2270-60-2	1257	1257					0.07	0.01
isobornyl acetate	125-12-2	1283	1280					0.25	0.03
NI			1295					0.05	0.01
NI			1320					0.04	0.01
δ-elemene	20307-84-0	1335	1332			0.19	0.01		
NI			1333					0.07	0.02
α-cubebene	17699-14-8	1348	1344					0.17	0.02
citronellyl acetate	150-84-5	1350	1349					0.06	0.02
NI			1360	0.13	0.03				
α-ylangene	14912-44-8	1373	1365					0.04	0.01
α-copaene	3856-25-5	1374	1370					0.34	0.15
β-patchoulene	514-51-2	1379	1375			5.14	0.94		
NI			1379	0.12	0.01				
NI			1379					0.01	0.00
β-elemene	515-13-9	1389	1386			0.77	0.08	2.28	0.10
NI			1390					0.04	0.01
sibirene	14029-18-6	1400	1395	0.07	0.01				
NI			1395					0.12	0.03
NI			1397	0.09	0.02				
NI			1397					0.06	0.01
cycloseychellene	52617-34-2	1406	1401			0.52	0.10		
dodecanal	112-54-9	1408	1403	0.14	0.02				
*(E)*-caryophyllene	87-44-5	1417	1413	1.36	0.28	4.23	0.57	2.37	0.19
NI			1422					0.18	0.03
γ-elemene	29873-99-2	1434	1427					0.57	0.02
NI			1432					0.13	0.02
NI			1440					0.15	0.03
α-himachalene	3853-83-6	1449	1442	0.02	0.00				
α-guaiene	3691-12-1	1437	1435			17.22	1.34		
NI			1444					0.09	0.02
6,9-guaiadiene	37839-64-8	1442	1444			0.67	0.05		
α-humulene	6753-98-6	1452	1447	0.68	0.06			1.72	0.07
α-patchoulene	560-32-7	1454	1449			8.17	2.08		
*(E)-*β-farnesene	18794-84-8	1454	1452					0.07	0.03
*allo*-aromadendrene	25246-27-9	1458	1453			3.29	1.48		
NI			1462			0.43	0.08		
NI			1456					0.13	0.01
NI			1468					0.18	0.02
NI			1471			0.53	0.03		
γ-muurolene	30021-74-0	1478	1471					0.82	0.03
NI			1474			0.22	0.02		
germacrene D	23986-74-5	1480	1476					4.01	0.22
NI			1477	0.13	0.01				
β-selinene	17066-67-0	1489	1480			0.17	0.02	0.25	0.05
NI			1483			0.35	0.03		
NI			1485					0.14	0.02
NI			1485			0.31	0.02		
NI			1488					0.05	0.01
NI			1488			0.12	0.02		
NI			1489	0.19	0.02				
NI			1490					0.01	0.00
α-muurolene	31983-22-9	1500	1494					0.35	0.01
aciphyllene	87745-31-1	1501	1493			3.29	0.28		
germacrene A	28387-44-2	1508	1498					0.16	0.03
γ-amorphene	6980-46-7	1495	1501					0.13	0.02
β-bisabolene	495-61-4	1505	1502	0.13	0.03				
*(Z)-*α-isabolene	70332-15-9	1506	1509	0.03	0.01				
α-bulnesene	3691-11-0	1509	1502			20.40	1.27		
γ-cadinene	39029-41-9	1513	1508					0.69	0.04
NI			1508			0.31	0.03		
7-epi-α-selinene	123123-37-5	1520	1512			0.36	0.02		
NI			1516			0.21	0.03		
δ-cadinene	483-76-1	1522	1518					1.77	0.40
*trans*-cadine-1,4-diene	38758-02-0	1533	1526					0.10	0.01
*(Z)-*γ-isabolene	13062-00-5	1514	1524	0.09	0.01				
NI			1527			0.42	0.02		
NI			1529			0.11	0.01		
NI			1529					0.02	0.01
NI			1532					0.03	0.01
NI			1535					0.30	0.05
NI			1543					0.04	0.01
elemol	639-99-6	1548	1544			0.01	0.00		
NI			1549			0.48	0.04		
germacrene B	15423-57-1	1559	1552					1.86	0.09
NI			1558					0.03	0.01
NI			1560			0.04	0.01		
NI			1563			0.11	0.01		
NI			1569			0.18	0.02		
NI			1571					0.06	0.01
NI			1577					0.07	0.02
caryophyllene oxide	1139-30-6	1582	1577			0.69	0.06		
NI			1582			0.56	0.04		
NI			1585			0.13	0.02		
NI			1603					0.03	0.01
NI			1603			0.23	0.03		
NI			1610			0.39	0.02		
NI			1614			0.29	0.03		
NI			1622			0.18	0.03		
NI			1622					0.05	0.01
NI			1626			0.22	0.02		
epi-α-cadinol	5937-11-1	1638	1635					0.11	0.01
NI			1640					0.04	0.01
NI			1640			0.21	0.03		
NI			1645			0.16	0.02		
α-cadinol	481-34-5	1652	1648					0.27	0.02
patchouli alcohol	5986-55-0	1656	1652			27.62	2.10		
NI			1661			0.14	0.02		
NI			1665			0.17	0.02		
NI			1671			0.06	0.01		
NI			1712			0.29	0.04		
Total identified				98.73		93.15		97.43	
Unidentified				1.27		6.85		2.57	
Total monoterpenes				96.21		0.41		79.29	
Total sesquiterpenes				2.52		92.74		18.14	
Total oxygenated monoterpenes				28.07		0		3.99	
Total oxygenated sesquiterpenes				0.14		28.31		0.38	

^a^ Constituents listed in order of elution on a non-polar DB-5 column; ^b^ Chemical Abstracts Service; ^c^ values obtained from Adams (2007); ^d^ retention index (RI) calculated by the retention time relative to that of a series of C_9_-C_30_ n-alkanes on a 30 m DB-5 capillary column; ^e^ %: area of compound relative to essential oil. ^f^ SD: standard deviation. NI: unidentified.

**Table 2:** Larvicidal activity of essential oils and formulations of leaves of *A. sibirica*, *J. communis* and *P. cablin* against larvae of the mosquito *A. aegypti*.

Essential Oils and Formulations	N ^a^	GL ^b^	LC_50_ (95% CI) ^c^ (LCL–UCL) ^d^ in ppm	LC_90_ (95% CI) ^c^ (LCL-UCL) ^d^ in ppm	*X* ^2 e^	Slope (SE)
*Abies sibirica* (A)	480	4	67.53 ± 2.03 (63.4–71.3)	122.24 ± 2.41 (109.23–145.76)	1.33	0.60
*Juniperus communis* (J)	420	4	92.45 ± 2.23 (87.7–96.4)	139.70 ± 2.21 (129.17–158.57)	0.99	0.94
*Pogostemon cablin* (P)	580	6	35.95 ± 2.03 (32.18–40.14)	126.71 ± 2.28 (102.49–169.57)	1.39	0.21
formulation (1:1:1) EO J + P + A	820	6	66.99 ± 0.90 (65.18–68.70)	94.39 ± 0.90 (90.24–100.05)	2.27	0.65
formulation (1:1) EO J + P	340	3	39.50 ± 1.34 (36.79–42.02)	70.07 ± 1.92 (63.87–78.92)	2.31	0.39
formulation (1:1) EO J + A	580	5	363.89 ± 19.43 (314.93–388.21)	669.78 ± 11.87 (577.91–980.27)	0.35	1.12
formulation (1:1) EO A + P	420	5	51.64 ± 2.40 (47.19–56.63)	122.67 ± 2.45 (103.76–155.62)	1.73	0.34

^a^ number of larvae used in the test; ^b^ degrees of freedom; ^c^ lethal concentration and confidence interval; calculations using statistical software StatPlus Pro 6.2.5.0 ^d^ and minimum (LCL) and maximum (UCL) lethal concentrations estimated by the ^e^ chi-squared statistic.

**Table 3:** Percentage of the number of eggs in the control and test, *p*-value, total number of eggs (N) and oviposition activity index (OAI) in the bioassays of the essential oils and formulations of the leaves of *A. sibirica, J. communis* and *P. cablin* and the compounds camphene, bornyl acetate and bornyl against female mosquitoes of *A. aegypti.*

Essential Oils and Formulations (Concentration)	OAI	Control (%)	Test (%)	*p*	N
Water-Tween × Water-Tween (control)	−0.07	54	46	0.213	4435
EO *A. sibirica (A),* 68 ppm	−0.41	71	29	0.000	5357
EO *J. communis* (J) 92 ppm	−0.31	66	34	0.009	4295
EO *P. cablin* (P) 36 ppm	−0.62	81	19	0.000	1785
Formulation (1:1:1) EO J + P + A, 67 ppm	−0.30	65	35	0.009	1659
Formulation (1:1) OE J + A, 100 ppm	−0.29	64	36	0.001	3131
Formulation (1:1) EO J + P, 40 ppm	−0.24	62	38	0.007	4232
Formulation (1:1) EO A + P, 51 ppm	−0.68	84	16	0.000	5787
camphene, 18.8 ppm	0.09	46	54	0.147	6981
bornyl acetate, 25 ppm	−0.14	57	43	0.576	4159
borneol, 2 ppm	0.10	45	55	0.364	3215

**Table 4:** LC_50_ values of some Brazilian plants found in the literature.

LC_50_ (ppm)	Species	Reference
67.53	*Abies sibirica* Ledeb	This study
60	*Ocimum gratissimum* L.	Cavalcanti et al., 2004 [50]
63	*Lippia sidoides* Cham.	
69	*Cymbopogon citratus* (DC.) Stapf.	
67	*Ocimum americanum* L.	
300	*Eugenia candolleana* DC.	Neves et al., 2017 [51]
97.7	*Cordia curassavica* (Jacq.) Roem. & Schult.	
313	*Alpinia zerumbet* (Pers.) Burtt & Smith	Cavalcantil et al., 2004 [50]
519	*Citrus limonia* Osbeck	
538	*Citrus sinensis* Osbeck	
433	*Syzygium jambolana* DC.	
261	*Hyptis suaveolens* Poit	
230	*Eugenia piauhiensis* Vellaff.	Dias et al., 2015 [52]
>100	*Myrcia erythroxylon* O. Berg	
292	*Psidium myrsinites DC.*	
251	*Siparuna camporum* (Tul.) A.DC.	
282	*Lippia gracilis* Schauer	
111.84	*Cymbopogon winterianus* Jowitt ex Bor	Cansian et al., 2023 [53]
92.45	*Juniperus communis* L	This study
144.69	*Baccharis reticularia* DC.	Rocha et al., 2022 [54]
152.0	*Croton Tetradenius* Baill.	
419.97	*Schinus terebinthifolia* Raddi	
138	*Carapa guianensis* Aublet	
99.40	*Commiphora leptophloeos* Leat	
35.95	*Pogostemon cablin*(Blanco) Benth	This study
37.88	*Piper gaudichaudianum* Kunth	Pereira et al., 2021 [48]
39.91	*Piper marginatum* Jacq.	
51.63	*Piper arboreum* Aubl.	
59.03	*Piper crassinervium* Kunth	

## Data Availability

All research data are already available in the article itself and in the supplementary material. However, additional information can be provided if necessary via email by the corresponding author.

## Supplementary Materials

The following supporting information can be downloaded at: https://www.mdpi.com/article/10.3390/molecules29245921/s1, Figure S1: Chromatogram of the oil essential of *Abies sibirica;* Figure S2: Chromatogram of the oil essential of *Juniperus communis;* Figure S3: Chromatogram of the oil essential of *Pogostemon cablin;* Table S1: Results obtained node rehearsal larvicide of oil of *Abies sibirica*; Table S2: Results obtained node rehearsal larvicide of oil of *Juniperus communis*; Table S3: Results obtained in rehearsal larvicide of oil of *Pogostemon cablin;* Table S4: Results obtained node rehearsal larvicide to the formulation 1:1 of the oils of *Juniperus communis* and *Pogostemon cablin;* Table S5: Results obtained node rehearsal larvicide to the formulation 1:1 of the oils of *Abies sibirica* and *Pogostemon cablin;* Table S6: Results obtained node rehearsal larvicide to the formulation 1:1 of the oils of *Abies sibirica* and *Juniperus communis;* Table S7: Results obtained in the larvicidal test for the formulation 1:1:1 of the oils of *Abies sibirica, Juniperus communis* and *Pogostemon cablin;* Table S8: Results obtained node rehearsal larvicide to the positive control temephos; Table S9: Results obtained node rehearsal of oviposition of oil of *Pogostemon cablin* at 36 ppm; Table S10: Results obtained node rehearsal of oviposition of oil from *Juniperus communis* at 92 ppm; Table S11: Results obtained node rehearsal of oviposition of oil of *Abies sibirica;* Table S12: Results obtained in the oviposition test for the formulation 1:1:1 of the oils of *Abies sibirica*, *Juniperus communis* and *Pogostemon cablin* at 67 ppm; Table S13: Results obtained in the oviposition test for the formulation 1:1 of the oils of *Juniperus communis* and *Pogostemon cablin* at 40 ppm; Table S14: Results obtained in the oviposition test for the formulation 1:1 of the oils of *Juniperus communis* and *Abies sibirica* at 100 ppm; Table S15: obtained node rehearsal of oviposition to the formulation 1:1 of the oils of *Abies sibirica* and *Pogostemon cablin* at 50 ppm; Table S16: Results obtained node rehearsal of oviposition to the solution of compound isolated bornyl at 2 ppm; Table S17: Results obtained node rehearsal of oviposition to the test of white Tween 80 in distilled water vs Tween 80 in distilled water; Table S18: Results obtained node rehearsal of oviposition to the solution of isolated compound camphene at 19 ppm; Table S19: Results obtained node rehearsal of oviposition to the solution of isolated compound bornyl acetate at 25 ppm.
