# Supplementary material for: Larvicidal and Oviposition Activity of Commercial Essential Oils of Abies sibirica Ledeb., Pogostemon cablin (Blanco) Benth., Juniperus communis L. and Their Combinations Against Aedes aegypti
Source: Molecules. 2024 Dec 15;29(24):5921. doi: 10.3390/molecules29245921 (PMC11677330; doi:10.3390/molecules29245921)
Supplement: Supplementary file 1 [file molecules-29-05921-s001.zip › molecules-3317782-supplementary.pdf]

Supplementary Material:

Characterization of the essential oils of *A. sibirica, J. communis and P. cablin:*

Figure S1-Chromatogram of the oil essential of *Abies sibirica*

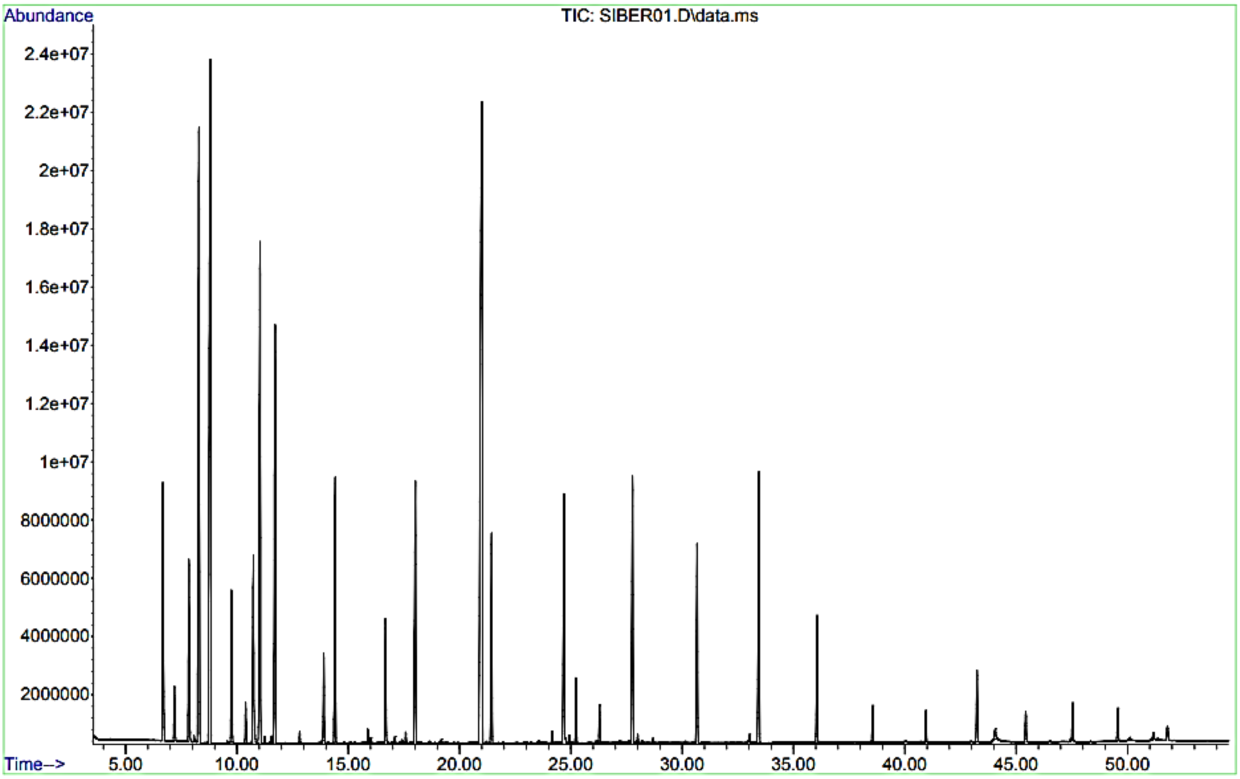

Compound (retention time, calculated RI, (retention index). ; Chemical Abstract Service, (CAS); unidentified (NI) = santene (6.649min; 882; 529-16-8); NI (7.170; 889; m/z: 40.0;53.0;67.0;79.0;93.0;107.1;122.1;207.1); tricyclene (7.818min; 917; 508-32-7); α-thujene (8.051min; 924; 2867-05-2); α-pinene (8.278min; 930; 80-56-8); camphene (8.799min; 944; 79-92-5); NI (9.547; 965; m/z: 40.0;51.0;65.0;77.0;91.0;130.0;119.1;134.1;207.0); β-pinene (9.739min; 970; 127-91-3); myrcene (10.375min; 988; 123-35-3); α-phellandrene (10.778min; 999; 99-83-2); δ-3-carene (11.024min; 1005; 13466-78-9); α-terpinene (11.235min; 1011; 99-86-5); ϱ-cymene (11.532min; 1019; 99-87-6); limonene (11.700min; 1024; 138-86-3); γ-terpinene (12.806min; 1053; 99-85-4); terpinolene (13.887min; 1082; 586-62-9); NI (14.419; 1097; m/z: 40.0;53.0;65.0;79.1;94.1;105.0;123.0;138.0;207.0); camphor (15.864min; 1137; 76-22-2); NI (15.995; 1140; m/z: 43.0;55.0;62.9;71.0;86.1;96.0;111.1;121.1;136.1;154.0); borneol (16.657min; 1159; 507-70-0); NI (17.392; 1179; m/z: 43.0;55.0;67.0;81.0;96.1;107.0;117.0;135.0;150.0;206.9); NI (17.571; 1184; m/z: 43.0;51.0;59.0;67.0;81.0;93.0;107.0;121.1;136.2); terpinen-4-ol (17.805min; 1171; 562-74-3); methyl ether thymol (19.172min; 1230; 1076-56-8); bornyl acetate (20.992min; 1283; 76-49-3); 2-undecanone (21.195min; 1289; 112-12-9); NI (23.530; 1360; m/z:

41.0;58.0;69.1;80.0;93.0;107.0;121.0;136.1;161.1;206.9); NI (24.152; 1379; m/z: 41.0;53.1;69.1;80.0;93.0;107.1;212.1;136.1;154.1); sibirene (24.681min; 1395; 14029-18-6); NI (24.748; 1397; m/z: 41.0;55.0;79.0;94.0;107.1;119.1;135.1;147.0;161.1;175.1;189.2;204.2); dodecanal (24.992min; 1403; 112-54-9); *(E)*-caryophyllene (25,210min; 1413; 87-44-5); α-himachalene (26.119min; 1442; 3853-83-6); α-humulene (26.274min; 1447; 6753-98-6); NI (27.194; 1477; m/z: 43.0;55.0;67.0;79.0;93.0;105.0;119.0;133.0;147.0;161.1;175.0;189.0;204.2); NI (27.584; 1489; m/z: 41.0;58.0;68.0;77.0;91.0;105.0;121.1;136.1;148.0;161.1;189.0;204.2); β-bisabolene (27.985min; 1502; 495-61-4); *(Z)*-α-isabolene (28.191min;1509; 70332-15-9); *(Z)*-γ- isabolene (28.669min; 1524; 13062-00-5).

Figure S2 - Chromatogram of the oil essential of *Juniperus communis*

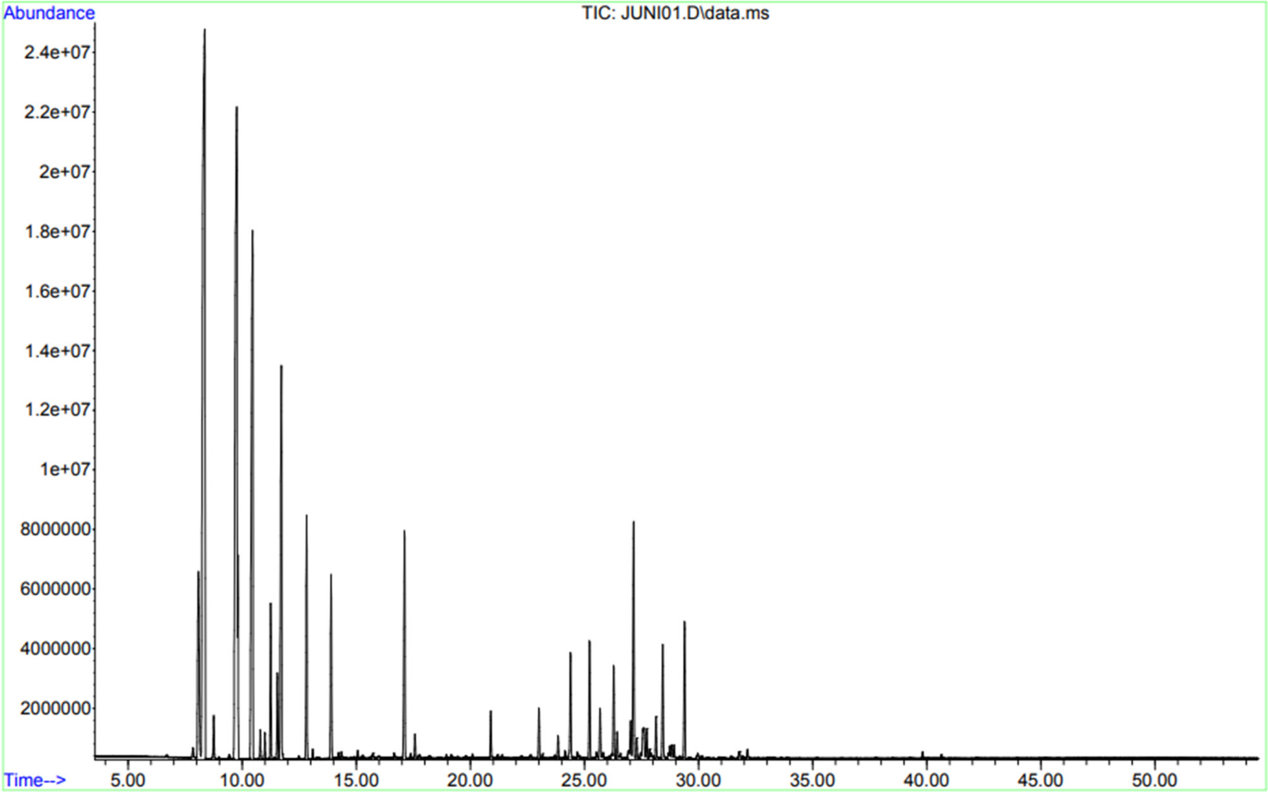

Compound (retention time, calculated RI, (retention index); Chemical Abstract Service, (CAS); unidentified (NI) = tricyclene (7.812min; 917; 508-32-7); α-thujene (8.044min; 924; 2867-05-2); α-pinene (8.328min; 930; 80-56-8); camphene (8.734min; 944; 79-92-5); NI (8.955; 949; m/z: 40.0;49.9;56.0;65.0;77.0;91.0;105.0;119.1;134.1); NI (9.417; 961; m/z: 41.0;51.0;65.0;77.0;91.1;105.1;119.1;134.1;207.0); sabinene (9.740min; 970; 3387-41-5); β-pinene (9.788min; 970; 127-91-3); myrcene (10.438min; 988; 123-35-3); α-phellandrene (10.770min; 999; 99-83-2); δ-3-carene (10.982min; 1005; 13466-78-9); α-terpinene (11.236min; 1011; 99-86-5); ϱ-cymene (11.530min; 1019; 99-87-6); 1,8-cineole (11.755min; 1025; 470-82-6); *(E)*-β-ocimene (12.473min;

1044; 3338-55-4); γ-terpinene (12.815min; 1053; 99-85-4); *cis*-sabinene hydrate (13.085min; 1061; 17699-16-0); terpinolene (13.891min; 1082; 586-62-9); *trans*-sabinene hydrate (14.214min; 1091; 15826-82-1); linalool (14.351min; 1095; 78-70-6); cis-ρ-menth-2-en-1-ol (15.062min; 1114; 29803-82-5); α-campholenal (15.254min; 1120; 4501-58-0); *trans*-sabinol (15.665min; 1131; 471-16-9); *trans*-limonene oxide (15.727min; 1133; 4959-35-7); NI (15.983; 1140; m/z: 43.0;55.0;71.0;79.1;96.0;110.1;121.1;136.0;152.0); borneol (16.651min; 1159; 507-70-0); terpinen-4-ol (17.144min; 1171; 562-74-3); NI (17.387; 1179; m/z: 35.9;43.0;51.0;65.0;77.0;91.0;105.1;117.1;126.9;135.1;150.1); NI (17.753; 1189; m/z: 41.0;53.0;67.0;79.0;91.0;107.1;121.1;131.0;139.1;151.9); α-terpineol (17.568min; 1184; 98-55-5); *cis*-piperitol (18.178min; 1201; 34350-53-3); citronellol (18.950min; 1223; 106-22-9); methyl NI (19.148; 1229; m/z: 43.0;54.0;65.0;77.0;91.0;105.0;119.1;134.1;149.1;164.1;194.0;207.0); NI (19.431; 1238; m/z: 43.0;53.0;67.0;85.1;95.0;106.0;119.1;134.1;150.1;206.9); NI (19.789; 1248; m/z: 43.0;55.0;67.0;82.0;93.1;110.0;121.1;136.1;152.1;206.9); citronellate (20.091min; 1257; 2270-60-2); isobornyl acetate (20.893min; 1280; 125-12-2); NI (21.383; 1295; m/z: 43.0;57.0;67.0;77.0;93.0;111.1;121.1;136.1;153.1;206.9); 2-undecanone (21.187min; 1289; 112-12-9); NI (22.222; 1320; m/z: 43.0;53.0;69.0;79.0;91.0;108.0;119.1;134.0;152.0); NI (22.631; 1333; m/z: 41.0;55.0;67.0;81.0;93.0;105.1;119.1;136.1147.0;161.4;176.1;189.1;204.1); α-cubebene (23.002min; 1344; 17699-14-8); citronellyl acetate (23.166min; 1349; 150-84-5); α-ylangene (23.695min; 1365; 14912-44-8); α-copaene (23.842min; 1370; 3856-25-5); NI (24.149; 1379; m/z: 41.0;53.1;69.1;81.0;93.0;107.1;121.1;136.1;147.1;161.1;175.1;189.1;204.2); β-elemene (24.388min; 1386; 515-13-9); NI (24.497; 1390; m/z: 40.0;55.0;65.9;81.0;91.0;105.1;119.1;133.1;151.0;161.1;182.1;204.1); NI (24.685; 1395; m/z: 41.0;55.0;67.0;79.0;91.0;105.1;119.1;133.0;147.1;161.2;175.1;189.2;204.2); NI (24.749; 1397; m/z: 41.0;55.0;67.0;79.1;94.1;107.1;119.1;135.1;147.1;161.1;175.1;189.2); *(E)*-caryophyllene (25.228min; 1413; 87-44-5); NI (25.525; 1422 ; m/z: 41.0;55.0;69.1;79.0;91.0;105.1;119.1;1333.1;147.0;161.2;175.1;189.1;204.2); γ-elemene (25.683min; 1427; 29873-99-2); NI (25.819; 1432; m/z: 41.0;55.0;67.0;79.0;91.1;105.1;121.1;133.0;147.1;161.2;175.2;189.2;204.2); NI (26.066; 1440; m/z: 42.0;55.0;65.0;81.0;91.0;105.1;119.1;132.9;145.0;161.1;189.1;204.2); NI (26.191; 1444; m/z: 41.0;55.0;69.0;81.1;91.0;105.1;119.1;133.0;145.1;161.2;189.1;204.2); α-humulene (26.282min; 1447; 6753-98-6); *(E)*-β-farnesene (26.440min; 1452; 18794-84-8); NI (26.569; 1456; m/z: 40;0;55.0;65.0;81.0;91.1;105.1;119.1;133.1;147.1;161.1;204.2 NI (26.919; 1468; m/z: 41.0;55.0;69.0;81.0;91.0;105.1;119.1;134.0;145.1;161.1;176.1;189.2;204.2); γ-muurolene (27.029min; 1471; 30021-74-0); germacrene D (27.169min; 1476; 23986-74-5); β-selinene (27.292min; 1480;

17066-67-0); NI (27.458 1485; m/z: 41.0;55.0;67.0;81.0;91.0;105.1;119.1;133.0;147.1;161.1;175.0;189 2;204.2); NI (27.560 1488; m/z: 41.1;55.0;67.0;79.0;93.1;105.1;119.1;133.1;147.1;161.1;175.2;189.2;204.2); NI (27.603 1490; m/z: 41.1;55.1;79.1;93.1;107.1;121.1;136.1;155.0;187.1;253.0); α-muurolene (27.729min; 1494; 31983-22-9); germacrene A (27.857min; 1498; 28387-44-2); γ-amorphene (27.935min; 1501; 6980-46-7); γ-cadinene (28.133min; 1508; 39029-41-9); δ-cadinene (28.433min; 1518; 483-76-1); *trans*-cadina-1,4-diene (28.669min; 1526; 38758-02-0); NI (28.741; 1529; m/z: 41.0;55.0;67.1;79.1;91.1;107.1;122.1;133.1;147.1;161.2;175.1;189.2;204.2); NI (28.830; 1532; m/z: 41.1;55.0;67.1;79.0;91.1;105.1;119.1;133.1;147.1;161.1;175.2;189.2;204.2); NI (28.926; 1535; m/z: 41.0;55.0;67.0;79.0;91.1;107.1;122.1;135.1;149.0;161.1;175.1;189.2;204.2;252.9); NI (29.165; 1543; m/z: 41.0;59.0;69.0;81.0;93.0;107.0;121.1;135.0;149.0;161.1;175.1;189.1;204.2); germacrene B (29.389min; 1552; 15423-57-1); NI (29.577; 1558; m/z: 40.0;55.0;69.0;81.0;93.0;107.0;119.0;132.9;147.1;157.1;175.1;189.2;204.2); NI (29.962; 1571; m/z: 43.0;65.0;79.0;91.0;105.1;119.1;132.0;145.1;159.1;174.1;187.1;205.2;220.1;252.9); NI (30.126; 1577; m/z: 41.0;55.0;69.0;79.0;93.0;107.1;121.1;133.0;147.0;161.1;177.0;189.2;204.1); NI (30.872; 1603; m/z: 43.0;55.0;67.0;79.0;93.0;109.1;121.0;138.1;148.9;159.0;177.1;204.1;220.1); NI (31.415; 1622; m/z: 41.0;55.0;69.0;81.0;93.0;105.1;119.1;133.0;147.0;161.1;179.1;204.2); epi-α-cadinol (31.784min; 1635; 5937-11-1); NI (31.918; 1640; m/z: 43.0;55.0;67.0;81.0;93.0;105.1;119.1;132.9;147.1;161.1;177.1;189.1;204.2;220.1); α-cadinol (32.140min; 1648; 481-34-5).

Figure S3- Chromatogram of the oil essential of *Pogostemon cablin*

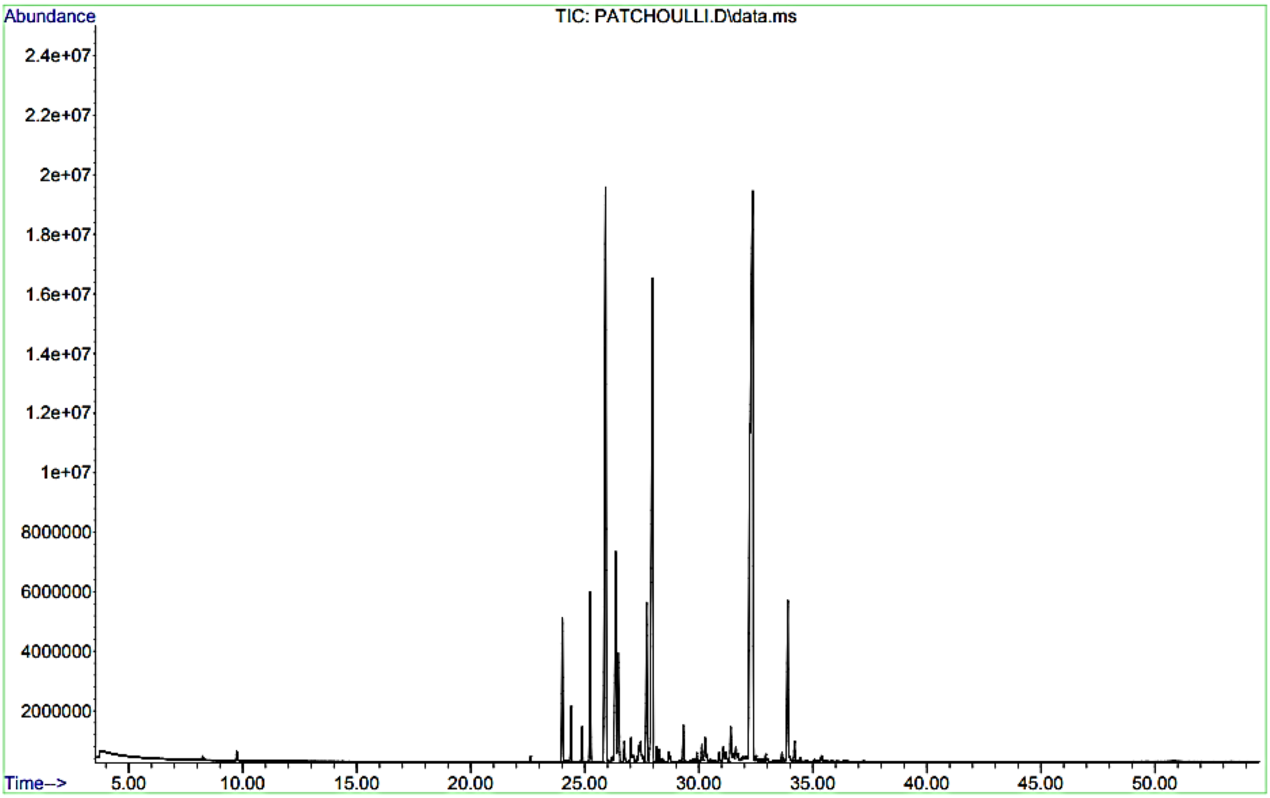

Compound (retention time, calculated RI, (retention index). ; Chemical Abstract Service, (CAS); unidentified (NI)= α-pinene (8.243min; 930; 80-56-8); β-pinene (9.738min; 970; 127-91-3); δ-elemene (22.620min; 132; 20307-84-0); β-patchoulene (24.017min; 1375; 514-51-2); β-elemene (24.394min; 1386; 515-13-9); cycloseychellene (24.878min; 1401; 52617-34-2); *(E)*-caryophyllene (25.242min; 1413; 87-44-5); α-guaiene (25.916min; 1435; 3691-12-1); 6,9-guaiadiene (26.186min; 1444; 37839-64-8); α-patchoulene (26.361min; 1449; 560-32-7); *allo*-aromadendrene (26.468min; 1453; 25246-27-9); NI (26.738;1462; *m/z:* 41.0;55.0;69.0;79.0;93.0;107.1;120.1;133.0;147.0;161.0;189.2;204.2); NI (27.025; 1471; *m/z:* 41.0;55.0;67.0;79.0;93.0;107.1;121.1;133.0;147.0;161.1;175.1;189.2;204.2); NI (27.111; 1474; *m/z:* 41.0;55.0;69.1;79.1;93.1;105.1;119.1;133.1;145.0;161.2;189.1;204.2); β-selinene (27.290min; 1480; 17066-67-0); NI (27.389; 1483; *m/z:* 41.0;55.0;67.0;79.0;93.0;107.1;121.1;133.1;147.1;161.1;175.2;189.2;204.2); NI (27.451; 1485; *m/z:* 41.0;55.1;69.1;81.1;91.1;105.1;119.1;133.1;147.1;161.1;175.1;189.2;204.2); NI (27.563; 1488; *m/z:* 40.0;55.0;67.0;81.1;93.1;109.1;121.1;133.1;147.1;162.1;175.1;189.2;204.2); aciphyllene (27.716min; 1493; 87745-31-1); α-bulnesene (27.982min; 1502; 3691-11-0); NI (28.153; 1508; *m/z:* 41.0;55.0;77.0;91.0;105.1;119.1;131.0;145.0;161.2;173.1;187.2;202.2;218.1;252.9); 7-epi-α-selinene (28.255min; 1512; 123123-37-5); NI (28.381; 1516; *m/z:* 41.0;53.0;65.0;79.0;91.0;105.1;119.1;131.0;145.0;159.1;173.1;187.2;202.2); NI (28.688; 1527; *m/z:*

41.0;55.0;65.0;77.0;91.0;105.1;119.1;131.1;145.1;161.1;173.1;187.2;202.2); NI (28.758; 1529; *m/z:* 41.0;53.0;65.0;77.0;91.0;105.1;119.1;133.1;146.1;159.1;173.1;187.1;202.2;253.0); elemol (29.175min; 1544; 639-99-6); NI (29.323; 1549; *m/z:* 41.0;55.0;67.0;79.1;91.1;107.1;122.1;137.0;149.0;163.2;178.1;191.2;206.2;253.0); NI (29.631; 1560; *m/z:* 40.0;55.0;69.1;81.0;93.0;107.1;121.1;133.0;147.0;161.1;177.1;189.1;204.1); NI (29.740; 1563; *m/z:* 41.0;55.0;67.0;81.0;93.0;107.1;121.1;135.0;145.0;162.1;177.1;187.1;202.1;220.1); NI (29.912; 1569; *m/z:* 43.0;55.0;67.0;81.0;93.0;107.1;119.1;133.0;147.0;161.1;173.1;187.2;202.2;220.2); caryophyllene oxide (30.137min; 1577; 1139-30-6); NI (30.276; 1582; m/z: 43.0;55.0;69.0;79.0;91.0;105.1;119.1;131.0;145.0;159.1;177.2;187.2;202.2;220.2); NI (30.357; 1585; m/z: 41.0;63.0;77.0;95.1;119.1;133.1;146.1;159.1;172.0;187.2;202.2;252.9); NI (30.885; 1603; m/z: 41.0;55.0;67.0;79.0;93.0;109.1;123.1;135.0;145.0;163.1;178.1;188.0;200.1;220.1); NI (31.081; 1610; m/z: 41.0;55.0;67.0;79.0;91.0;105.1;123.1;134.1;145.0;159.1;173.1;187.1;202.2;220.2); NI (31.185 ;1614; m/z: 43.0;55.0;67.0;79.1;91.1;105.1;119.1;131.0;145.0;159.1;174.1;187.2;202.2;220.1;253.0); NI (31.402 ;1622; m/z: 41.0;55.0;67.0;81.0;95.0;109.1;125.1;137.0;147.1;161.2;175.2;189.2;204.2;222.1); NI(31.510;1626;m/z:43.0;55.0;67.1;79.0;91.0;105.1;119.1;134.0;147.1;159.1;177.1;190.0;202.2;220.1;252.8);NI(31.923;1640;m/z:41.0;55.0;69.1;83.0;95.1;111.1;123.1;137.0;149.0;161.1;175.2;189.1;207.2;222.1;253.0);NI(32.060;1645;m/z:40.0;55.0;69.0;83.0;97.0;111.1;122.1;133.1;147.1;165.1;179.1;193.1;204.2;222.2); patchouli alcohol (32.363min; 1652; 5986-55-0); NI (32.513; 1661; *m/z:* 40.0;55.1;69.0;91.1;107.1;120.,;133.0;147.1;161.1;175.1189.2;203.2;218.2;281.10); NI (32.629; 1665; *m/z:* 43.0;69.0;93.1;119.1;145.0;162.0;187.1;205.1;281.1;355.0); NI (32.782; 1671; *m/z:* 40.0;55.0;69.0;84.1;107.0;121.1;136.1;149.0;161.1;176.1;191.1;205.1;220.1;238.2); NI (33.905; 1712; *m/z:* 43.0;55.0;69.0;85.1;97.0;111.0;125.1;140.1;153.1;168.1;181.1;195.1;209.1;224.2);

Larvicidal activity: Tables A1 to A8 refer to the experimental results of the oviposition bioassays, the total number of dead larvae and the total number of larvae used at each concentration and the mortality percentage for each concentration range used in determining the LC_{50}.

Table S1: Results obtained node rehearsal larvicide of oil of *Abies sibirica*

Concentration (ppm)	Total no. of dead	Total no. of larvae	% mortality
50	23	80	28.75
60	29	80	36.25
70	43	80	53.75
80	49	80	61.25
90	60	80	75.00
100	65	80	81.25

Table S2- Results obtained node rehearsal larvicide of oil of *Juniperus communis*

Concentration (ppm)	Total no. of dead	Total no. of larvae	% mortality
80	28	80	35.0
90	37	80	46.3
100	45	80	56.3
110	42	60	70.0
120	47	60	78.3
130	53	60	88.3

Table S3- Results obtained in rehearsal larvicide of oil of *Pogostemon cablin*

Concentration (ppm)	Total no. of dead	Total no. of larvae	% mortality
10	7	80	8.8
20	20	80	25.0
25	31	80	38.8
30	28	60	46.7
50	49	80	61.3
60	43	60	71.7
70	44	60	73.3
80	63	80	78.8

Table S4- Results obtained node rehearsal larvicide to the formulation 1:1of the oils of *Juniperus communis* and *Pogostemon cablin*

Concentration (ppm)	Total no. of dead	Total no. of larvae	% mortality
20	9	80	11.3
30	21	80	26.3
40	50	100	50.0
50	43	60	71.7
60	51	60	85.0
80	57	60	95.0
100	59	60	98.3

Table S5- Results obtained node rehearsal larvicide to the formulation 1:1 of the oils of *Abies sibirica* and *Pogostemon cablin*

Concentration (ppm)	Total no. of dead	Total no. of larvae	% mortality
20	6	60	10.0
30	13	60	21.7
40	18	60	30.0
50	29	60	48.3
60	34	60	56.7
80	47	60	78.3
100	50	60	83.3

Table S6- Results obtained node rehearsal larvicide to the formulation 1:1of the oils of *Abies sibirica* and *Juniperus communis*

Concentration (ppm)	Total no. of dead	Total no. of larvae	% mortality
350	29	60	48.33
375	32	60	53.33
400	56	100	56.00
425	63	100	63.00
450	53	80	66.25
475	71	100	71.00
500	61	80	76.25

Table S7- Results obtained in the larvicidal test for the formulation 1:1:1 of the oils of *Abies sibirica, Juniperus communis* and *Pogostemon cablin*

Concentration (ppm)	Total no. of dead	Total no. of larvae	% mortality
90	89	100	89.00
85	50	60	83.33
80	119	160	74.38
75	38	60	63.33
70	86	160	53.75
65	29	60	48.33
60	32	100	32.00
50	19	120	15.83

Table S8- Results obtained node rehearsal larvicide to thepositive control temephos

Concentration (ppb)	Total no. of dead	Total no. of larvae	%
1	5	60	8.33
1.75	20	80	25.00
3.25	47	100	47.00
3.5	50	100	50.00
3.75	68	120	56.67
4	38	60	63.33

Oviposition activity: Tables S9 to S19 refer to the experimental data of the number of eggs collected in each bioassay at LC_{50} for each oviposition site, in the control and in the test for each cage.

Table S9 – Results obtained node rehearsal of oviposition of oil of *Pogostemon cablin* at 36 ppm

Cage	Control		Test		
	No. of eggs	%	No. of eggs	%	Total no. of eggs
1	423	80	109	20	532
2	57	48	62	52	119
3	238	100	0	0	238
4	118	90	13	10	131

5	97	97	3	3	100
6	327	91	33	9	360
7	108	100	0	0	108
8	82	42	115	58	197
Total of eggs	1450	81	335	19	1785

Table S10 – Results obtained node rehearsal of oviposition of oil from *Juniperus communis* at 92 ppm

Cage	Control		Test		
	No. of eggs	%	No. of eggs	%	Total no. of eggs
1	308	57	236	43	544
2	88	22	315	78	403
3	383	69	176	31	559
4	294	74	105	26	399
5	502	85	92	15	594
6	340	65	186	35	526
7	491	73	186	27	677
8	414	70	179	30	593
Total of eggs	2820	66	1475	34	4295

Table S11 – Results obtained node rehearsal of oviposition of oil of *Abies sibirica*

Cage	Control		Test		
	No. of eggs	%	No. of eggs	%	Total no. of eggs
1	293	64	164	36	457
2	415	71	168	29	583
3	552	80	140	20	692
4	628	88	89	12	717
5	662	79	174	21	836
6	481	63	282	37	763
7	559	74	194	26	753
8	194	35	362	65	556
Total of eggs	3784	71	1573	29	5357

Table S12 – Results obtained in the oviposition test for the formulation 1:1:1 of the oils of *Abies sibirica*, *Juniperus communis* and *Pogostemon cablin* at 67 ppm

Cage	Control		Test		
	No. of eggs	%	No. of eggs	%	Total no. of eggs
1	0	0	62	100	62
2	243	83	50	17	293
3	139	56	111	44	250
4	132	66	68	34	200
5	47	92	4	8	51
6	138	88	19	12	157
7	209	95	11	5	220
8	168	39	258	61	426

Total of eggs	1076	65	583	35	1659

Table S13 – Results obtained in the oviposition test for the formulation1:1 of the oils of *Juniperus communis* and *Pogostemon cablin* at 40 ppm

Cage	Control		Test		
	No. of eggs	%	No. of eggs	%	Total no. of eggs
1	37	14	227	86	264
2	190	35	358	65	548
3	199	35	366	65	565
4	227	44	284	56	511
5	165	23	556	77	721
6	260	60	173	40	433
7	144	27	394	73	538
8	384	59	268	41	652
Total of eggs	1606	38	2626	62	4232

Table S14 – Results obtained in the oviposition test for the formulation1:1 of the oils of *Juniperus communis* and *Abies sibirica* at 100 ppm

Cage	Control		Test		
	No. of eggs	%	No. of eggs	%	Total no. of eggs
1	146	70	62	30	208
2	279	63	167	37	446
3	394	63	234	37	628
4	113	42	158	58	271
5	436	67	215	33	651
6	200	75	68	25	268
7	136	50	137	50	273
8	311	81	75	19	386
Total of eggs	2015	64	1116	36	3131

Table S15-obtained node rehearsal of oviposition to the formulation1:1 of the oils of *Abies sibirica* and *Pogostemon cablin* at 50 ppm

Cage	Control		Test		
	No. of eggs	%	No. of eggs	%	Total no. of eggs
1	650	77	194	23	844
2	651	82	146	18	797
3	142	87	21	13	163
4	1007	89	127	11	1134
5	625	75	208	25	833
6	371	73	136	27	507
7	560	98	14	2	574
8	869	93	66	7	935
Total of eggs	4875	84	912	16	5787

Table S16- Results obtained node rehearsal of oviposition to the solution ofcompound isolated bornyl at 2 ppm

Cage	Control		Test		
	No. of eggs	%	No. of eggs	%	Total no. of eggs
1	192	71	80	29	272
2	292	51	275	49	567
3	126	26	363	74	489
4	256	67	124	33	380
5	71	25	211	75	282
6	244	63	141	37	385
7	175	37	293	63	468
8	83	22	289	78	372
Total of eggs	1439	45	1776	55	3215

Table S17- Results obtained node rehearsal of oviposition to the test ofwhite Tween 80 in distilled water vs Tween 80 in distilled water

Cage	Control		Test		
	No. of eggs	%	No. of eggs	%	Total no. of eggs
1	208	29	516	71	724
2	358	48	395	52	753
3	115	46	133	54	248
4	268	61	169	39	437
5	415	70	175	30	590
6	347	53	310	47	657
7	213	60	142	40	355
8	458	68	213	32	671
Total of eggs	2382	54	2053	46	4435

Table S18- Results obtained node rehearsal of oviposition to the solution of isolated compound camphene at 19 ppm

Cage	Control		Test		
	No. of eggs	%	No. of eggs	%	Total no. of eggs
1	538	50	540	50	1078
2	411	51	398	49	809
3	49	9	518	91	567
4	219	27	595	73	814
5	395	55	324	45	719
6	406	44	512	56	918
7	654	56	508	44	1162
8	516	56	398	44	914
Total of eggs	3188	46	3793	54	6981

Table S19- Results obtained node rehearsal of oviposition to the solution of isolated compound bornyl acetate at 25 ppm

Cage	Control		Test		
	No. of eggs	%	No. of eggs	%	Total no. of eggs
1	241	43	314	57	555
2	204	54	172	46	376
3	96	48	106	52	202
4	188	66	98	34	286
5	449	59	318	41	767
6	657	73	249	27	906
7	499	75	162	25	661
8	27	7	379	93	406
Total of eggs	2361	57	1798	43	4159
